# Renal Health Improvement in Diabetes through Microbiome Modulation of the Gut–Kidney Axis with Biotics: A Systematic and Narrative Review of Randomized Controlled Trials

**DOI:** 10.3390/ijms232314838

**Published:** 2022-11-27

**Authors:** Pradipta Paul, Ridhima Kaul, Ali Chaari

**Affiliations:** 1 Medical Education Division, Weill Cornell Medicine-Qatar, Cornell University, Qatar Foundation—Education City, Doha P.O. Box 24144, Qatar; 2 Premedical Division, Weill Cornell Medicine-Qatar, Cornell University, Qatar Foundation—Education City, Doha P.O. Box 24144, Qatar

**Keywords:** gut flora, microbiota, nutraceutical, short-chain fatty acids, inflammation, uremic toxins, bile acids, nephropathy

## Abstract

Diabetes mellitus is the most common endocrine disorder worldwide, with over 20% of patients ultimately developing diabetic kidney disease (DKD), a complex nephropathic complication that is a leading cause of end-stage renal disease. Various clinical trials have utilized probiotics, prebiotics, and synbiotics to attempt to positively modulate the gut microbiome via the gut–kidney axis, but consensus is limited. We conducted a multi-database systematic review to investigate the effect of probiotics, prebiotics, and synbiotics on various biomarkers of renal health in diabetes, based on studies published through 10 April 2022. Adhering to the Cochrane Collaboration and Preferred Reporting Items for Systematic Reviews and Meta-Analyses (PRISMA) guidelines, relevant articles were systematically screened and extracted by independent reviewers; subsequently, results were systematically compiled, analyzed, and expanded through a narrative discussion. A total of 16 publications encompassing 903 diabetic individuals met the inclusion criteria. Our findings show that some studies report statistically significant changes in common renal markers, such as serum creatinine, estimated glomerular filtration rate, blood urea nitrogen/urea, microalbuminuria, and uric acid, but not on serum albumin, sodium, potassium, phosphorous, or total urine protein. Interestingly, these nutraceuticals seem to increase serum uric acid concentrations, an inflammatory marker usually associated with decreased renal health. We found that probiotics from the *Lactobacillus* and *Bifidobacterium* families were the most investigated, followed by *Streptococcus thermophilus*. Prebiotics including inulin, galacto-oligosaccharide, and resistant dextrin were also examined. The single-species probiotic soymilk formulation of *Lactobacillus plantarum* A7 possessed effects on multiple renal biomarkers in DKD patients without adverse events. We further investigated the optimum nutraceutical formulation, discussed findings from prior studies, described the gut–kidney axis in diabetes and DKD, and finally commented on some possible mechanisms of action of these nutraceuticals on renal health in diabetics. Although probiotics, prebiotics, and synbiotics have shown some potential in ameliorating renal health degradation in diabetes via gut–kidney axis crosstalk, larger and more convincing trials with focused objectives and next-generation nutraceutical formulations are required to investigate their possible role as adjunct therapy in such patients.

## 1. Introduction

Diabetes mellitus is one of the most prevalent chronic diseases around the globe, currently affecting more than 537 million adults worldwide and spreading at accelerated rates [1]. Along with enormous physical and psychological burdens on patients, it is responsible for large proportions of healthcare expenditures and is a major public health concern [2]. Its most common form is type 2 diabetes (T2D), an endocrine disorder characterized by chronic hyperglycemia, impaired pancreatic islet β-cell function, and increased insulin resistance. T2D often leads to debilitating macrovascular complications, such as cardiovascular disease (CVD) [3], as well as microvascular complications such as retinopathy, neuropathy, and nephropathy [4]. Afflicting 20–40% of diabetic patients, diabetic nephropathy (DN), or diabetic kidney disease (DKD), is a complex and multifactorial complication of diabetes involving hyperglycemia, atherosclerosis, obesity, dyslipidemia, hypertension, and increased glomerular pressure [5,6,7]. DKD is also one of the leading causes of end-stage renal disease (ESRD). Macro-proteinuria is present in 50% of newly diagnosed T2D patients within 20 years, while microalbuminuria and reduced glomerular filtration rates (GFRs) were shown to be present in 38% and 29% of newly diagnosed patients within 15 years of follow-up, respectively [8]. With respect to socioeconomics, there is a consistent and proportional pattern between a state’s gross national income per capita and its use of more recent and effective diabetic medications, such as sodium glucose transport-2 (SGLT-2) inhibitors and glucagon-like peptide-1 (GLP-1) agonists [9]. Despite a wide library of anti-diabetic drugs to choose from, low-income and middle-income country (LMIC) diabetics require cost reductions in order to afford more recent, effective, and tolerable treatments, and may be unable to wait for costs to decrease with time [10]. Given that four in five diabetics reside in LMICs, there is an urgent need for inexpensive, yet effective treatment programs and regimens that slow or inhibit the progression of nephropathy and reduce associated morbidity and mortality [1].

The role of the gut microbiome or intestinal flora has become a much-discussed topic in fields associated with multiple gastrointestinal, inflammatory, oncological, and endocrine disorders, including diabetes [11,12,13,14,15,16,17]. While the association between the gut microbiome and the systemic pathophysiology of diabetes is complex and multifactorial, it is widely acknowledged that a dysbiosis in the gut microbiota is a common pathophysiological feature [18]. Secondary metagenomic analyses in diabetic children have highlighted the protective effects of microbial metabolites, while other studies have referenced their role in microbiota–gut–brain communication [19,20]. Given that diet, weight control, and lifestyle modification are the leading non-pharmacological management options in diabetes [21], dietary supplementation with gut microbiome dysbiosis-targeting nutraceuticals are an attractive, cost-effective, and promising option that may serve as an adjunct therapy in ameliorating renal status in diabetics [22]. As per the most recent consensus statements of the International Scientific Association for Probiotics and Prebiotics (ISAPP), probiotics are defined as “live microorganisms which when administered in adequate amounts confer a health benefit on the host” [23]; prebiotics as “a substrate that is selectively utilized by host microorganisms conferring a health benefit” [24]; and lastly, synbiotics as “a mixture comprising live microorganisms and substrate(s) selectively utilized by host microorganisms that confers a health benefit on the host” [25]. Previously, we have shown that such nutraceuticals may improve inflammation, oxidative stress, liver damage, glycemia, and insulinemia in T2D patients [26,27,28]. Recently, researchers have also successfully investigated their role in obesity-related kidney disease and hypertension [29,30]. Although other studies have investigated the effect of multiple nutraceuticals on metabolic biomarkers and outcomes in participants with selected renal diseases, there is limited research in the literature providing a qualitative yet comparative summary of all three supplementation types in all diabetic patients [31,32,33,34,35]. This systematic review of randomized controlled trials (RCTs) aims to summarize and analyze whether probiotic, prebiotic, and synbiotic supplementation produces a clinically significant, beneficial effect on biomarkers of renal function in patients with diabetes.

## 2. Methods

### 2.1. Study Protocol

We conducted a systematic review following the Cochrane Collaboration Handbook guidelines [36] and reported results following the Preferred Reporting Items for Systematic Reviews and Meta-Analysis (PRISMA) [37]. 

### 2.2. Data Sources and Search Strategy

We conducted database searches across PubMed, Scopus, Web of Science, Embase, Cochrane, ClinicalTrials.org, and ProQuest Dissertations & Theses in June 2020, and then ran an updated search on April 10, 2022. The extensive search strategy and elements required to replicate the search are provided in Supplementary Table S1. Briefly, the string used on PubMed is as follows: (“Probiotics”[MeSH Terms] OR “probiotics”[Title/Abstract] OR “probiotic”[Title/Abstract] OR “Prebiotics”[MeSH Terms] OR “prebiotic”[Title/Abstract] OR “prebiotics”[Title/Abstract] OR “Synbiotics”[MeSH Terms] OR “synbiotics”[Title/Abstract] OR “synbiotic”[Title/Abstract] OR “symbiotic”[Title/Abstract] OR “symbiotics”[Title/Abstract] OR “gastrointestinal microbiome”[MeSH Terms] OR “gut microbiome”[Title/Abstract] OR “gut flora”[Title/Abstract]) AND (“diabetes mellitus, type 2”[MeSH Terms] OR “T2D”[Title/Abstract] OR “type 2 diabetes”[Title/Abstract]), limited to clinical studies.

### 2.3. Eligibility Criteria and Screening

We included all clinical trials or RCTs investigating the effect of microbiome-modulating nutraceuticals, such as probiotics, prebiotics, and synbiotics, on various biomarkers of renal function and health in patients with diabetes. These markers included serum creatinine (Cr), blood urea nitrogen (BUN), microalbuminuria, uric acid, serum phosphorous, sodium, potassium, proteinuria, eGFR, and/or other renal biomarkers. Studies of any duration, published at any time, and involving adult participants of any age, sex, ethnicity, and from any region worldwide were included. We did not discriminate against studies including populations with other comorbidities or conditions, provided that diabetes was a major focus or comprised most of the included participants. We excluded reviews, conferences, abstracts and proceedings, editorials and non-clinical papers, animal studies, and studies in languages other than English. We further excluded studies focusing on other diseases or gestational diabetes, other biomarkers, and those administering non-bacterial pro/synbiotics. All references were imported into Covidence where duplicates were removed and at least two reviewers screened titles, abstracts, and full texts systematically. Conflicts were resolved by consensus.

### 2.4. Data Extraction

Once the selection process was complete, we performed data extraction using pre-piloted tables in Microsoft Word. Extracted variables are elucidated in Supplementary Table S2. Daily pro/prebiotic dosage, if not exclusively specified, was reported according to nutraceutical formulation and daily frequency. We extracted means ± SDs, median and interquartile ranges (IQRs), and mean differences (MDs) with 95% confidence intervals (CIs) for values of renal biomarkers at baseline, end-of-trial, and change over time for both intervention and control groups. *p*-values were also extracted whenever reported. Units were maintained as reported in the primary publication during extraction, and later converted during analysis and discussion.

### 2.5. Risk of Bias Assessment

We used the Cochrane risk-of-bias tool version 2 (RoB2) in order to score and report the risk of bias (ROB) associated with individual publications [36]. Some of the pre-piloted factors used to assess ROB included randomization process, allocation concealment, participant recruitment, deviations from intended intervention, missing outcome data, outcome measurement, and selection of reported results. Studies were classified as having either some concerns, high ROB, or low ROB with respect to judgement regarding the above factors.

## 3. Results

### 3.1. Search Results

**Figure 1:**
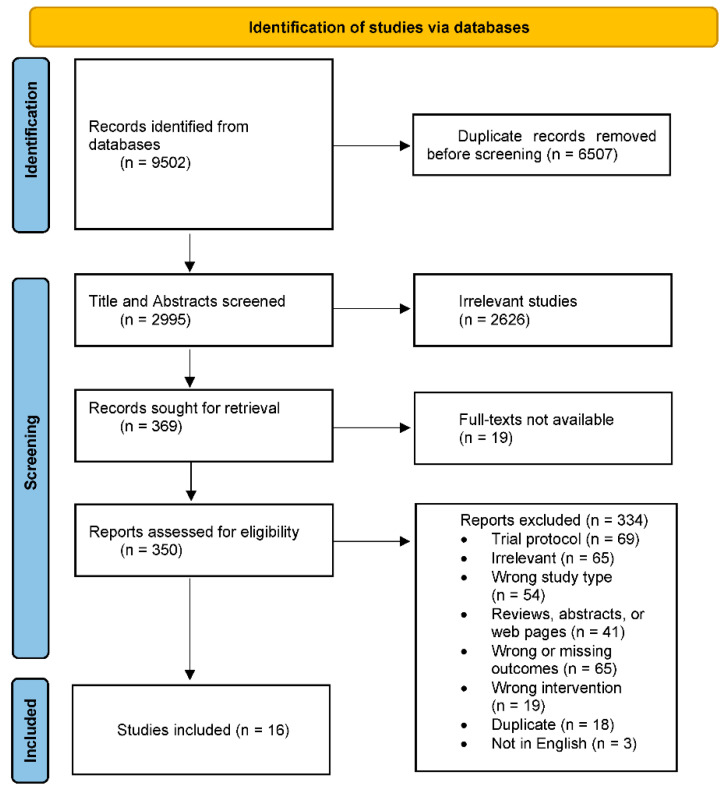
Flow diagram of search strategy and included studies and trial comparisons.

The initial electronic search yielded 9502 records from all seven databases, of which 6507 were deemed duplicates during the import process and were removed by Covidence. Title and abstract screening of the remaining 2995 records yielded 369 potential full texts to be screened. Of these, 16 publications were found to be eligible under the inclusion criteria and were included in this systematic review. The various reasons for excluding the remaining studies have been summarized in Figure 1. Of the 16 studies included [38,39,40,41,42,43,44,45,46,47,48,49,50,51,52,53], three records [38,39,46] were found to be related to the same clinical trial and involved the same participants, hence the three studies were discussed as one RCT or trial. Additionally, one study [47] investigated two distinct intervention groups linked to one control group, which we deemed equivalent to two RCTs. Lastly, another study [44] was designed in a crossover manner, yielding two distinct intervention groups from the same participants who also acted as the control; however, only combined results have been reported, yielding one RCT. This systematic review therefore includes 15 RCTs from 16 distinct publications.

### 3.2. Trial Characteristics

Detailed characteristics of each included trial are provided in Table 1. This review encompasses data from 903 distinct diabetic RCT participants (414 control, 427 intervention, and 62 serving as both in a crossover fashion). With regards to inclusionary comorbidities, five (33.3%) of the included trials specified only T2D, whereas two (13.3%) reported each of the following: T2D with overweight, T2D with abdominal obesity, T2D with DN, DN only, and diabetic hemodialysis. Most (n = 6, 40.0%) included trials were investigations of multispecies probiotics, followed by single species probiotics (n = 4, 26.7%), prebiotics (n = 3, 20.0%), and lastly single species (n = 1, 6.7%) and multispecies synbiotics (n = 1, 6.7%). Of the 11 pro/synbiotic administering trials that reported dosage information, the median daily bacterial dose was 4.0 × 10^9^ colony-forming units per day (CFU/d; IQR: 1.4 × 10^9^–9.0 × 10^9^; range: 5 × 10^6^–6 × 10^10^), whereas among the four pre/synbiotic administering trials that reported dosage information, the median daily prebiotic dose was 10 g per day (g/d; IQR: 7.7–10; range: 1.08–10). While many forms of supplemental matrix or carrier agents were reported, the most popular delivery was via capsules (n = 8, 53.3%), followed by other forms, such as syrup, honey, milk, bread, powder, or sachets. With respect to participant characteristics, the mean control/placebo and intervention group ages were 55.4 and 55.8 years, respectively, with a mean baseline body mass index (BMI) of 28.9 kg/m^2^ for control/placebo and 29.0 kg/m^2^ for the intervention group (BMI information was unavailable for one study [40]). Most trials were published in 2017 (median: 2017; range: 2013–2021) and lasted 12 weeks (median: 12; range: 4–12). Of the 15 RCTs, the majority of trials were based in Iran (n = 9, 60.0%), whereas the two RCTs (13.3%) by Mobini et al. from the same study [47] were based in Sweden. Of the remaining studies, one trial (6.7%) was based in each of Japan, China, Malaysia, and Egypt.

### 3.3. Risk of Bias Assessment

A summary of ROB associated with studies assessed using the Cochrane collaboration RoB2 tool has been provided in Supplementary Figure S1, while assessment of individual studies with respect to each factor is shown in Supplementary Figure S2. Overall, 11 studies (68.8%) were found to have low ROB, 4 (25.0%) had some concerns, while only 1 (6.3%) had a high ROB. Similar figures were true with respect to only the randomization process. One study (6.3%) had high ROB in participant recruitment, 12 (75.0%) had low ROB and 3 (18.8%) had some concerns. With respect to deviations from intended intervention, the majority of studies (13; 81.3%) had a low ROB, while only 3 (18.8%) had some concerns, with none having high ROB. With respect to outcome measurement, 14 studies (87.5%) had low ROB, while 1 (6.3%) study had some concerns and high ROB. With respect to both missing outcome data and selection of reported results factors, 15 (93.8%) of studies had low ROB, with only one (6.3%) having high ROB.

### 3.4. Effect on Serum Creatinine (Cr)

Eight RCTs investigated the effect of pro/pre/synbiotic supplementation on serum creatinine (Cr) levels (Table 1). Of these, only two reported significant changes compared to control/placebo, both being reductions in serum Cr following probiotic administration. Abbasi et al. [38] showed that, in a group of 40 T2D-DN patients, 8-week supplementation with probiotic soy milk with a daily dose of 4 × 10^9^ CFU *Lactobacillus plantarum* A7 significantly reduced serum Cr levels (Δ from baseline: −0.17 ± 0.11 mg/dL), compared to both baseline values (*p* < 0.05) and changes observed in a control group (adjusted *p* < 0.0001) receiving conventional soy milk. In another cohort of 60 DN patients (56 T2D/4 T1D), Mafi et al. [52] showed that 12-week supplementation with a multispecies probiotic containing *Lactobacillus acidophilus* ZT-L1, *Bifidobacterium bifidum* ZT-B1, *L. reuteri* ZT-Lre, and *L. fermentum* ZT-L3, amounting to a total probiotic dosage of 8 × 10^9^ CFU/d, reduced serum Cr by −0.2 ± 0.3 mg/dL from baseline, a change that was statistically significant compared to changes observed in a control group receiving starch. While some of the other studies also reported decreases, such as that of −0.1 ± 0.5 mg/dL from baseline in a cohort of 60 DN patients, reported by Mazruei Arani et al., this is not found to be statistically significant (*p* = 0.09) [48].

### 3.5. Effect on Estimated Glomerular Filtration Rate (eGFR)

Seven RCTs investigated the effect of pro/pre/synbiotic supplementation on estimated glomerular filtration rate (eGFR) levels (Table 1). Of these, only two reported significant changes compared to the control/placebo, both being an increase in eGFR after probiotic administration. In the previously described RCT, Abbasi et al. [38] also showed a significant increase of 15.9 ± 10.8 mL·min^−1^ (1.73 m^2^)^−1^ in eGFR from baseline. This change was also significant compared to both baseline values (*p* < 0.05) and changes in control/placebo group (adjusted *p* < 0.0001). Secondly, Mafi et al. [52] also showed that in the previously described RCT, multispecies probiotics increased eGFR by 8.3 ± 17.3 mL/min from baseline, a change that was considered significant upon comparison to the one observed in the control group (*p* = 0.001). Furthermore, while both Soleimani et al. [53] and Farhangi et al. [41] also reported slight increases in eGFR following probiotic and prebiotic administration, respectively, these were not found to be statistically significant compared to control/placebo group (*p* = 0.74 [adjusted] and 0.65, respectively).

### 3.6. Effect on Urea or Blood Urea Nitrogen (BUN)

Six RCTs investigated the effect of pro/pre/synbiotic supplementation on urea or blood urea nitrogen (BUN) levels (Table 1). Of these, only two reported significant changes compared to control/placebo, with both studies reporting a decrease in urea/BUN after multispecies probiotic administration. In a large cohort of 136 T2D patients based in Malaysia, Firouzi et al. [50] showed that 12-week administration of microbial cell preparations containing 6.0 × 10^10^ CFU/d *Lactobacillus acidophilus*, *L. casei*, *L. lactis*, *Bifidobacterium bifidum*, *B. longum*, and *B. infantis* mixed in water was responsible for a significant reduction in urea (from 4.26 ± 1.29 at baseline to 4.04 ± 1.04 mmol/L at end-of-trial) in the intervention group, compared to an increase in the control/placebo group (*p* < 0.05). Using a slightly different formulation containing 8 × 10^9^ CFU/d *Lactobacillus acidophilus* ZT-L1, *Bifidobacterium bifidum* ZT-B1, *L. reuteri* ZT-Lre, and *L. fermentum* ZT-L3, the previously described study by Mafi et al., 2018 [52] reported changes of −3.5 ± 5.8 mg/dL in BUN from baseline in the intervention group, a significant change compared to the control/placebo (*p* = 0.03). While reductions in mean BUN have been reported by at least two other studies [48,53], these were not found to be statistically significant.

### 3.7. Effect on Urine Albumin/Creatinine Ratio (Alb/Cr)

Five RCTs investigated the effect of pro/pre/synbiotic supplementation on the urine albumin/creatinine ratio (Alb/Cr) or microalbuminuria levels (Table 1). Of these, three reported significant reductions compared to the control/placebo, whereas the other two, both from the study by Mobini et al. [47], showed no effects. In the previously described study by Abbasi et al. [38], the authors reported that administration of a single species probiotic resulted in a change of −16.5 ± 12.2 mg/g from baseline urine Alb/Cr, compared to −5.7 ± 15.04 mg/g in those receiving control, a difference in change that was found to be significant (*p* = 0.03). In another cohort of 76 T2D-DN patients based in China, Jiang et al. [51] showed that, following administration of multispecies probiotic supplements containing 3.2 × 10^9^ CFU/d *Bifidobacterium bifidum*, *Lactobacillus acidophilus*, and *Streptococcus thermophilus*, urine Alb/Cr decreased from 101.60 ± 22.17 to 67.53 ± 20.11 mg/g, compared to significantly different (*p* < 0.05) end-of-trial mean of 87.71 ± 23.01 mg/g in the control group which had a similar baseline value. In another cohort of 70 T2D patients from Iran studied by Ebrahimi et al. [45], administration of a once-per-day synbiotic capsule containing 500 mg of *Lactobacillus* family, *Bifidobacterium* family, *Streptococcus thermophilus*, fructo-oligosaccharides (FOS), B group vitamins, lactose, maltodextrin, magnesium saturate, and talc resulted in a change of −10.44 ± 35.26 mg/g from baseline, compared to an increase of 18.31 ± 46.78 mg/g in the control/placebo group; the difference between these changes was found to be significant (*p* < 0.0001).

### 3.8. Effect on Uric Acid

Three RCTs investigated the effect of pro/pre/synbiotic supplementation on urine uric acid levels (Table 1). Of these, only one reported a significant change, which was following synbiotic supplementation. Asemi et al. [44] showed that 6-week supplementation of a synbiotic containing 2.7 × 10^8^ CFU/d heat-resistant *Lactobacillus sporogenes*, 1.08 g/d inulin, isomalt, sorbitol, and stevia resulted in an increase of 0.7 ± 0.2 mg/dL in uric acid levels, which when compared to the change of −0.1 ± 0.3 mg/dL in the control/placebo group receiving the same material without synbiotic, showed a significant overall effect (*p* = 0.03). Although similar increases from baseline were reported in another study by Asemi et al. (Δ: +0.8 ± 0.27 mg/dL; *p* = 0.008) [49] and Farhangi et al. (MD: 1.85 mg/dL [95% CI: 0.91; 1.24], *p* < 0.05) [42], neither of these results were found to be statistically significant when compared to the corresponding change in control/placebo groups.

### 3.9. Effect on Serum Sodium, Potassium, and Phosphorus

Two RCTs investigated the effect of multispecies probiotic supplementation on serum sodium and potassium levels (Table 1), and both reported no significant change over time. In the previously described study among T2D patients by Firouzi et al. [50], the authors showed that although both sodium (from 138.5 ± 2.2 to 138.1 ± 3.5 mmol/L) and potassium (from 4.42 ± 0.30 to 4.35 ± 0.31 mmol/L) levels decreased slightly from baseline following probiotic use over 12 weeks, the change was not significant (*p* = 0.235 and 0.164, respectively) compared to control/placebo. Similar results were also produced by Soleimani et al. [53] in their investigation among diabetic hemodialysis patients, where probiotics prevented a slight increase in serum sodium and slightly decreased serum potassium; however, neither of these changes were significant (adjusted *p* = 0.07 and 0.18, respectively). Two other RCTs investigated the effect of pro/prebiotic supplementation on serum phosphorous levels (Table 1) and reported no significant change over time. Abbasi et al. [38] reported a minor change of −0.14 ± 0.10 mg/dL from baseline following probiotic milk consumption; however, when compared to the control receiving conventional soy milk, this change was not significant (*p* = 0.106). Furthermore, Farhangi et al. [41] reported almost no change in serum phosphorous following two months of oligofructose-enriched chicory inulin consumption in a cohort of 49 overweight T2D patients. Hence, current evidence shows that probiotics do not appear to significantly improve serum ions of renal significance.

### 3.10. Effect on Serum Albumin

Two RCTs investigated the effect of multispecies probiotic supplementation on serum albumin (Table 1), with both reporting slight increases following 12-week regimens in diabetic hemodialysis patients; however, neither of these changes were significant compared to control/placebo. Soleimani et al. [53] reported a change of 0.1 ± 0.3 g/dL from baseline; however, this was not significant compared to both baseline (*p* = 0.45) and the change in control groups (adjusted *p* = 0.48). Mosbah et al. [40] also reported a similar change among 30 patients following a 12-week regimen of capsules containing 5 × 10^6^ *Lactobacillus delbrueckii* and *L. fermentum*, erythropoietin stimulating agents (ESA), and antidiabetic agents, compared to a control group (n = 30) receiving only placebo capsules, ESA, and antidiabetic agents. Although the mean change of 0.1 g/dL was significant compared to baseline (*p* = 0.039), the authors did not clarify whether it was significant compared to control.

### 3.11. Effect on Other Renal Biomarkers

Few RCTs investigated the effect of pro/pre/synbiotics on renal health by utilizing novel or lesser-utilized biomarkers (Table 1). Mafi et al. [52] showed that although a change of −14.3 ± 40.1 mg/day in urine total protein was observed among their DN participants, this change was not statistically significant (*p* = 0.10). Secondly, in their RCTs investigating the effect of probiotic soy milk among T2D-DN participants, Abbasi et al. [39] and Miraghajani et al. [46] reported on multiple promising markers of renal function. A change of −49.18 ± 48.22 pg/mL in serum interluekiin-18 (IL-18), compared to that of −9.03 ± 18.65 pg/mL among controls receiving conventional soy milk, was found be significant (adjusted *p* = 0.002). Similar significant results were reported with respect to relative change compared to control/placebo group with respect to progranulin (PGRN; adjusted =0.01), soluble tumor necrosis factor receptor (sTNFR1; adjusted *p* = 0.03), serum sialic acid (SSA; *p* = 0.001), neutrophil gelatinase-associated lipocalin (NGAL; adjusted *p* = 0.05), and cystatin C (Cys-C; adjusted *p* = 0.01) (Table 1).

## 4. Discussion

### 4.1. Main Findings

This systematic review pooled data from 15 RCTs across 16 distinct publications to compare and analyze the effect of probiotics, prebiotics, and synbiotics on various biomarkers of renal health among 903 participants with diabetes. To our knowledge, this is the only comprehensive review of all three types of microbiome-modulating nutraceuticals among diabetics. Our results show that pro/pre/synbiotic supplementation has the potential to ameliorate imbalances in various reno-metabolic markers, such as serum Cr, eGFR, BUN, Alb/Cr ratio, IL-18, PGRN, SSA, sTNFR1, NGAL, and Cys-C, under appropriate intervention duration, target population, and nutraceutical formulation; however, no change in serum albumin, sodium, potassium, phosphorous, or urine total protein was observed among any of the reviewed studies, whereas one study reported a statistically significant and potentially harmful increase in uric acid.

### 4.2. Is there an Optimum Nutraceutical Formulation?

While most of the pro/synbiotic-utilizing studies in this review included various strain and species combinations of the *Lactobacillus* and *Bifidobacterium* genus, a few studies also included *Streptococcus thermophilus* in their formulations. Dosage varied between trials (ranging from 5 × 10^6^ to 6 × 10^10^ CFU/d) and did not follow any particular patterns. Furthermore, all prebiotic administering trials utilized 10 g/d of inulin, galacto-oligosaccharide (GOS), or resistant dextrin, whereas one synbiotic trial reported using 1.08 g/d of inulin. While most nutraceutical interventions were supplied as independent capsules, a minority were supplemented by other forms, such as syrup, honey, milk, bread, powder, or sachets. Of the various combinations of interventions used in the 15 RCTs, we find that the single-species probiotic soy milk formulation consisting of 4 × 10^9^ CFU/d *Lactobacillus plantarum* A7 [38,39,46] produced significant effects on T2D-DN patients without adverse events, modifying the most renal biomarkers, including serum Cr, eGFR, urine Alb/Cr, serum IL-18, sTNR1, Cys-C, and PGRN. However, it should be noted that this observation stems from one RCT with data on separate markers provided across three publications [38,39,46]. As no other study has investigated supplementation using *Lactobacillus plantarum* A7 individually, in combination, or via a soy milk medium, it is difficult to ascertain the exact mechanism behind its multifactorial benefits, or whether this is attributed to the specific probiotic strain/species, the effects of bioactive compounds in soy [54,55,56], or a third, synergistic mechanism [57].

It has been hypothesized that probiotic soy milk yields its effects through inflammation reduction, pro-inflammatory cytokines, and oxidative stress, thereby attenuating glomerular injuries and tubulointerstitial lesions, while further increasing bioavailability of beneficial flavonoids [58,59,60,61]. Additionally, it has also been shown that probiotics translocate harmful gut microbiome bacteria [62] as well as their bacterial products, such as trimethylamine N-oxide, p-cresol, and indoxyl sulfate, which have been shown to independently damage podocytes and renal tubules via various complex mechanisms [63,64,65]. In addition to possessing cell-surface hydrophobicity and adhesion properties, *Lactobacillus plantarum* A7 has proven to be tolerant to various acidic and bile environments, making it an ideal probiotic to survive in and influence the gut [66]. In diabetic rats, Babashahi et al. [67] report that adding *Lactobacillus plantarum* A7 probiotic to soy milk improves metabolic outcomes such as fasting glucose and lipid profile; however, when added with *Cuminum cyminum*, the effects were even greater. This finding needs to be investigated in human trials.

With respect to alternative promising formulations, the multispecies probiotic combination of *Lactobacillus acidophilus* ZT-L1, *Bifidobacterium bifidum* ZT-B1, *L. reuteri* ZT-Lre, and *Lactobacillus fermentum* ZT-L3 in capsules induced significant changes in BUN, serum Cr, and eGFR among patients with DN [52]. Although three studies [41,42,43] investigated the effects of prebiotics on various renal biomarkers, no promising effects were seen. Furthermore, resistant dextrin was found to increase uric acid [42], while GOS decreased eGFR [43], both significantly compared to baseline, but not when compared to control. These findings are yet to be reproduced and explained mechanistically. Lastly, synbiotics show moderate promise to ameliorate dysregulated renal biomarkers such as uric acid and the urine Alb/Cr ratio, but only two studies [47,51] investigated the effect of synbiotics on renal health. To both fully understand and further investigate the potential synergism in synbiotics, more high quality, transparent, and comprehensive investigations that study all or most of the renal biomarkers over greater intervention durations are required.

### 4.3. Findings from Previous Reviews

Although ours is the first comprehensive analysis of the effect that pro/pre/synbiotics have on various renal parameters in diabetics, previous systematic reviews and meta-analyses have independently and exclusively investigated the effect of pro/pre/synbiotics on renal markers in only T2D, DN/DKD, or other diseases. In a study by Abdollahi et al. [5], pooled results from six pro/synbiotic administering RCTs revealed significant changes of −0.10 mg/dL (95% CI: −0.20; −0.00) in serum Cr among T2D participants; however, the study found no significant effects on eGFR, Alb/Cr ratio, or BUN. Similar results were found by Tarrahi et al. [68] from their analysis of seven trials administering probiotics in DN patients, revealing significant changes of −0.18 mg/dL (95% CI: −0.26; −0.09) in serum Cr, but no change in BUN or GFR. Contrarily, in their recent meta-analysis of DKD patients, Dai et al. [34] reported that probiotics improved multiple biomarkers of renal injury, including serum Cr, BUN, Cys-C, the Alb/Cr ratio, and sodium, but this analysis stemmed from the exclusion of a study of elderly DN patients by [69] due to language-based exclusion criteria. This, coupled with the low number of trials per marker, made the single study that reported significant benefits on serum Cr, BUN, Cys-C, and urine total protein [69] a large influence on the overall pooled results. In an earlier review, Wang et al. [70] reached similar conclusions to Dai et al. [34], where the team reported that probiotics were beneficial in improving renal function in DN patients by increasing eGFR and decreasing both serum Cr and BUN; however, this contrasts with the previously described findings of Tarrahi et al. [68] concerning eGFR and BUN. Less recent reviews [71,72] included fewer trials and did not show promise with respect to any renal biomarker, likely owing to smaller sample sizes. The success of more recent trials can be thus attributed to more effective formulations or trial characteristics. Interestingly, a review by Firouzi et al. [73] revealed that based on available trials on both healthy and diseased patients, pro/pre/synbiotics improved BUN, urea, and uric acid levels. Overall, although these findings are consistent with findings from previous reviews, these conclusions are based on a smaller number of studies, each with small sample sizes, and thus must be interpreted with caution.

### 4.4. Gut–Kidney Axis in Diabetes and Diabetic Kidney Disease

**Figure 2:**
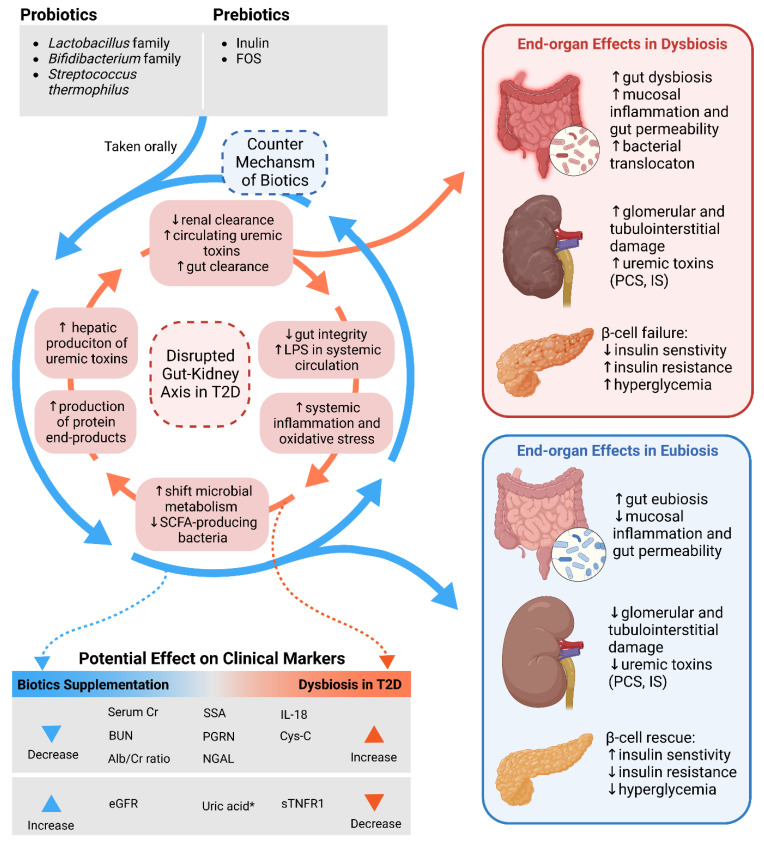
Schematic representing the disrupted gut–kidney axis in diabetes (in red) versus the potential counter mechanism (in blue) of biotic nutraceuticals (probiotics, prebiotics, and their combinations), in addition to their respective effects on end-organs and clinical (renal) biomarkers.

A complex ecosystem containing trillions of bacteria belonging to thousands to species, the large intestinal “gut” microbiota plays an important, bidirectional role in human health and disease [74]. In a symbiotic relationship with the host, the microbiome produces several important and beneficial metabolites and secondary bile acids via metabolism of dietary macronutrients, such as proteins and carbohydrates. Furthermore, the gut microbiome protects against harmful pathogens via maintenance of the gut immune barrier, competition for limited resources, and reduced translocation of microbial compounds, such as harmful bacterial endotoxins. There is accumulating evidence suggesting that a healthy and balanced gut–host relationship is crucial for maintenance of host health, and that dysbiosis of this bidirectional crosstalk is often involved in the pathogenesis of various metabolic diseases, including diabetes [16] (Figure 2), which has been established by landmark papers in the field [75,76]. Lower microbiome diversity and lack of butyrate-producing bacteria have been shown to be associated with onset and development of T2D [77], and bacterial richness has important associations even in other metabolic diseases [77,78]. This is apparent in chronic kidney disease (CKD), where retention or build-up of toxic uremic solutes of microbial origin in the circulation triggers fibrotic, apoptotic, and inflammatory pathways, ultimately leading to exacerbation of intestinal dysbiosis and permeability, contributing to renal failure [79].

Previously known as DN, DKD is defined as diabetes with microalbuminuria (Alb/Cr ratio ≥ 30 mg/g) and/or impaired eGFR (<60 mL/min/1.73 m^2^). It develops in 30–40% of diabetics, and is the most prominent predictor of premature mortality in such patients [8,80]. It has been postulated that DKD is characterized by a multi-pathway pathogenesis based on hemodynamic, metabolic, and inflammatory changes [81]. Hemodynamically, there is increased efferent arteriolar vasoconstriction arising from activation of the renin–angiotensin–aldosterone system (RAAS) and subsequent increased angiotensin II levels leading to increased blood pressure. Hyperglycemia in diabetes activates glycolysis, in turn upregulating various sub-pathways which lead to onset or worsening of various aspects of DKD [82]. A chronically activated immune system and state of low-grade inflammation further contributes to renal damage, mediated by various cytokines and cellular cascades [83]. This is supported by findings of alteration in composition and function of microbiota of DKD patients compared to T2D patients without signs of DKD, highlighting that kidney manifestations may have salient independent features that play important roles in the pathogenesis of DKD independent of T2D [84]. In fact, recent findings have revealed the role of the metagenome in blood pressure: microbiome-derived short-chain fatty acids (SCFAs) have been shown to mediate blood pressure via olfactory receptor 78 (Olfr78) and Gpr4. Alteration of SCFAs, and by extension dysbiosis of the gut microbiome, can stimulate hypertension through Olfr78, renin secretion, and modulation of peripheral resistance [85].

Abundance of bacteria of Proteobacteria phylum in the gut of DKD patients aligns with previous findings that show Proteobacteria-dominated microbiomes were characteristically associated with elevated inflammation, a key finding in the pathogenesis of DKD [84,86]. In the same study [84], the non-DKD group had higher levels of *Bacteroides*, which are known to produce SCFAs from dietary fiber. SCFAs are being independently investigated for their therapeutic potential in systemic inflammatory, immune, and metabolic diseases, and have been shown to play important roles in various cellular signaling pathways [87,88]. In another recent study, Tao et al. [89] revealed that in T2D patients with biopsy-proven DKD, there is a stark reduction in gut microbiota richness compared to age- and sex-matched healthy controls. This was accompanied with an observation that *Actinobacteria*, Bifidobacteriaceae, and *Prevotella* genus were more abundant in patients with diabetes, and Coriobacteriaceae were enriched in those with DKD, revealing their particular contribution to DKD. Higher levels of *Escherichia-Shigella* and lower levels of *Prevotella* were found to be particularly capable of differentiating DKD group individuals, further revealing characteristic associations. More interestingly, the group reported that clinical reno-metabolic parameters were also associated with patterns in gut microbiota. For instance, *Fusobacteria* was negatively correlated with fasting glucose, and *Firmicutes* was negatively associated with levels of fasting glucose, HbA1c, and urinary Alb/Cr ratio, whereas *Verrucomicrobiota* was significantly correlated with eGFR.

In another recent metagenomic study [90], abundancies of butyrate-producing bacteria such as *Clostridium*, *Eubacterium*, and *Roseburia intestinalis*, and that of potential probiotics such as *Lachnospira* and *Intestinibacter*, were significantly reduced in both T2D and DKD group fecal samples compared to age-, sex-, and BMI-matched healthy controls. DKD patient’s fecal samples were further characterized by increased *Bacteroides stercoris*. *Clostridium sp*. 26_22, and *L. mucosae* were inversely predictive of serum Cr and LDL in DKD patients, whereas in T2D patients, *Lachnospira* and *R. bicirculans* were positively correlated with serum Cr and HbA1c, respectively, and *Prevotellamassilia* and *P. timonensis* were negatively correlated with HbA1c [90]. The study also promotes the potential of gut microbiota to serve as a diagnostic marker for DKD, with *Pseudomonadales*, *F. varium*, and *Prevotella* sp. MSX73 being significantly associated with T2DM and DN based on mean decrease Gini (MDG)-based random forest analysis, with some being more accurate predictors of diagnosis compared to traditional clinical indices. These findings have been documented in earlier, smaller human studies [91] as well as experimental investigations among mice [92], thus enforcing their interstudy validity.

In a recent metabolomics study by Kikuchi et al. [93], the gut microbiome-derived uremic toxin metabolite phenyl sulfate was shown to function as a marker of DKD progression and was correlated with the urine Alb/Cr ratio in a cohort of 363 diabetic patients following 2 years of follow-up. The mechanism of action behind this correlation is thought to be mediated by its podocyte damage and proinflammatory and profibrotic effects [93]. In one animal study, researchers found that gut microbiome dysbiosis was responsible for activation of G protein-coupled receptor 43 (GPR43) and contributed to albuminuria in DN [94]. Inhibiting the microbiota-specific enzyme tyrosine phenol-lyase, which plays an important part in the cascade for synthesis of phenyl sulfate, was shown to reduce albuminuria and serum Cr [93], providing a connection in the gut–kidney axis.

The role of the diseased gut microbiome in production of uremic toxins that aggravate vascular and renal toxicity is established in the literature. Fueled by dietary limitations, altered intestinal transit time, and chronic uremic status in CKD, a shift from saccharolytic to proteolytic fermentation in the host microbiota leads to decreased renal clearance and simultaneous increase in colonic production [95,96,97,98]. This also explains the increased levels of circulating urea in CKD patients due to growth of bacteria with urease and uricase in response to the selective pressure caused by an influx of urea and uremic toxins into the GI lumen [99]. Thus, as the microbially derived metabolites are found to mediate systemic effects on host immune physiology, it is not surprising to see why modulating the gut microbiome is thus an attractive subject of research. Although recent pharmacological advancements, such as SGLT2 inhibitors and conventional renin–angiotensin–aldosterone system (RAAS) inhibitors, have successfully reduced diabetes-associated CVD and morbidity and ameliorated CKD progression in T2D patients, there still exists considerable risk for ESRD progression, paving the way for newer therapeutics or adjuncts such as microbiome-modulating nutraceuticals [98].

### 4.5. Mechanisms of Action of Microbiome-Modulating Nutraceuticals

Dietary modification is one of the initial therapeutic strategies to prevent or mitigate clinical manifestations in various chronic metabolic diseases. Changes in diet to include higher fiber or lower protein have been traditionally utilized to achieve reno-metabolic outcomes such as eGFR and serum Cr [100,101]. One such dietary modification is the inclusion of probiotics, prebiotics, or synbiotics to promote healthy and diverse gut microbiomes. They are believed to possess anti-inflammatory, anti-oxidative, and other gut-modulatory activities that can help ameliorate the reno-metabolic imbalance observed in T2D and DKD (Figure 2) [102]. In addition to the studies that investigate the effect of pro/pre/synbiotics on T2D that have been analyzed in this review, studies in the literature have investigated their effect on other renal conditions, such as CKD, ESRD, and hemodialysis [103]. Across these studies, nutraceuticals have shown potential to reduce BUN, serum uric acid, dimethylamine, and nitroso-dimethylamine, while improving blood levels of nitro-dimethylamine [102,104,105,106]. Of particular interest are commercially abundant probiotics of the genus *Lactobacillus*, *Bifidobacterium*, and to a lesser degree, *Streptococcus*, which have been widely investigated.

Recent dietary recommendations for renal disease patients include promotion of gut health through increased prevalence of beneficial bacteria such as *Bifidobacterium, Lactobacillus*, and *Eubacterium* spp., and lowering the presence of proteolytic bacteria, thus confirming the potential to directly influence the microbiome through dietary changes [61]. One proposed mechanism is the ability of lactic acid bacteria (LAB), such as *Bifidobacterium* and *Lactobacillus*, to prevent proliferation of aerobic bacteria in the gut, in turn promoting a balanced microbiome that modulates urea levels [50,73]. Furthermore, the urease activity of some special probiotics such as *Bacteroides* species may also improve urea degradation and thus decrease urea levels [60,107].

Another postulated mechanism that considers the impaired intestinal microbiota in renal disease is the modulation of gut pH by LABs [72]. In CKD, impaired gut microbiota is characterized by aerobic bacteria such as *Escherichia coli*, thus leading to increased urea and pH levels. Supplementation using LABs decreases pH via fermentation of carbohydrates and competitive exclusion of harmful pre-existing aerobes [58,59]. In the earlier discussed RCT by Abbasi et al. [38,39,46], probiotic soy milk containing *Lactobacillus plantarum* A7 could reduce IL-18 levels, whose serum concentration has been independently shown to correlate with DKD development and is utilized as a clinical marker for vulnerability and progression to advanced renal disease [108,109,110,111,112]. This may be more due to the effects of soy and its associated mechanism with flavonoids rather than the probiotic, as has been shown following soy nut consumption in postmenopausal women with metabolic syndrome [113]. However, in a recent study by Wang et al. [114], stage 3–5 CKD patients supplemented with *Lactobacillus acidophilus*, *Bifidobacterium longum* spp. *Infantis*, and *B. bifidum* probiotics were shown to have reduced IL-18, along with reduced IL-6, TNF-α, and slower deterioration of eGFR following six months of treatment. Stool microbiota presence of *B. bifidum* and *B. breve* was also significantly increased. These results highlight the potential of probiotics to improve systemic innate immunity involvement along with pro-inflammatory cytokines in renal disease.

In DKD, damage of the vascular renal endothelial cells owing to hyperglycemia leads to increased SSA, thus serving as a biomarker for renal health, as has been shown across various populations over the years [115,116,117,118,119]. Probiotic soymilk with *Lactobacillus plantarum* A7 has been shown to decrease SSA levels compared to soymilk alone in DN patients, perhaps by reducing renal microvascular complications, glomerular damage, or tubulointerstitial fibrosis due to its antioxidant effects. There is some evidence that *Lactobacillus plantarum* A7 also decreases cystatin-C, a recently proposed biomarker that, when used to calculate eGFR, serves as a better predictor of all-cause mortality in a multiethnic elderly cohort, in comparison with serum Cr [38,39,46,120]. One of the key mechanisms by which probiotics cater their health benefits is via production of SCFA metabolites such as acetate, propionate, and butyrate. In acute kidney injury (AKI) rat models, Oliveira et al. [121] have revealed the potential of these three SCFAs to improve renal dysfunction caused by injury. This process is thought to be mediated by lower local and systemic inflammation, oxidative cellular stress, cell infiltration/activation, and apoptosis [121]. SCFAs were also found to reduce hypoxia in kidney epithelial cells through the promotion of mitochondrial biogenesis, and ultimately had better outcomes overall following AKI.

Microbiome-modulating nutraceuticals have been investigated critically for not only their potential to promote a healthy microbiome, but also to directly reduce levels of uremic toxins, which act as key mediators in renal disease. In a double-blind RCT, stage 3–4 CKD patients receiving synbiotics were shown to have reduced p-cresol (a uremic toxin) concentrations, thus possibly serving to delay progression to ESRD in such patients [122]. This potential to reduce circulating levels of uremic toxins such as indoxyl sulfate and p-cresol sulfate has also been reported following use of prebiotics such as resistant starch for just six weeks in hemodialysis patients [123]. Prebiotics support the healthy growth and proliferation of the normal gastrointestinal microbiota, with resistant starches, oligosaccharides, and inulin being some of the most investigated and commercially available prebiotics [124]. Their potential lies in their basic nature to resist digestion by intestinal juices and are thus fermented by the microbiota in the gut, leading to increased metabolite production that have their own beneficial effects [125].

Prebiotics such as FOS have been shown to increase butyrate production and promote growth of butyrate-producing bacteria such as *Bifidobacterium pseudolongum Lactobacillus, Coprococcus*, and *Enterococcus* [126]. Recent studies have also shown their potential to reduce serum and total levels of uremic toxins such as p-cresyl sulfate in non-diabetic CKD patients. Resistant starch has been investigated with good success for its potential to reduce polyuria and disruption of vitamin D homeostasis in Type 1 diabetic rats via rescue of renal megalin-mediated endocytosis [127]. In other animal studies, it was shown to improve gut microbiome and metabolomic profiles, and delay progression to CKD [128,129]. However, their potential use in DKD to improve renal function still needs to be examined by high-quality RCTs. Prebiotic co-administration with probiotics in the form of synbiotics aims to synergistically improve outcomes by promoting further growth and increased reach along the gut, as previously described [122]. Although various probiotic/prebiotic combinations have been investigated against numerous diseases in the literature, it is still debated whether certain probiotics “prefer” specific prebiotics in order to produce specific SCFAs and not others [130].

In the recent literature, *Bifidobacteria* has been shown to metabolize FOS and inulin-type fructans in order to produce acetate and lactate, respectively, and *Lactobacilli* has been shown to utilize inulin preferentially for production of lactate. These are mediated by the presence of specific enzyme gene clusters [131,132,133,134,135]. Fecal microbiota transplantation (FMT) is another newly emerging form of microbiome modulation that has the potential to restore intestinal structure, ameliorate inflammation, and serve therapeutically in diabetes [136]. FMT from healthy donors has been shown to improve podocyte-involved glomerular injury in experimental studies with diabetic rats, in a process mediated by restoration of the AMPKα activity [94]. Lastly, postbiotics, or functional metabolites of bacteria such as the SCFAs acetate, propionate, and butyrate, are emerging with promising therapeutic effects against high blood pressure, hyperglycemia, hyperlipidemia, and even for the prevention and treatment of DN and other kidney diseases [29,137,138,139].

### 4.6. Limitations

This study has a few limitations. Firstly, as the extraction phase was non-blinded to the reviewers, this could introduce some bias. A limitation concerning the search strategy is that since prebiotics are rather less researched and discussed in the literature, it may be that some substances are not yet identified as prebiotics formally. In this case, this study may not have captured all sources of prebiotic administration among diabetics [140,141]. Due to potentially different mechanisms of action of non-bacterial probiotics, such as the fungus *Saccharomyces boulardii*, we have limited the current review of microbiome-modulating probiotics on bacteria; this should be further differentiated in future studies [142,143]. As we have discussed, there were considerable differences in the intervention characteristics among the trials studied; these come in the form of differences in nutraceutical type, mode of delivery, formulations, number, species type (pro/synbiotics), varying intervention durations, and different populations. This variety made it difficult to analyze the effects of a particular strain or prebiotic across different research studies. Moreover, most, if not all, of the included trials have very small sample sizes and were further concentrated in regions of the Eastern Mediterranean, particularly Iran, thus this should invite caution during interpretation of the generalizability of these findings. Lastly, we did not consider variables such as adverse effect profiles and direct changes in gut microbiome composition, largely due to limited availability of such information across trials; nevertheless, these are important factors that should be investigated by future trials and closely analyzed by upcoming reviews.

## 5. Conclusions and Recommendations

In this systemic review, we have reviewed clinical trials that investigated probiotics, prebiotics, and synbiotics to improve renal health in diabetics. Various clinical renal biomarkers, such as serum creatinine, GFR, Alb/Cr ratio, BUN, and others, were used as proxy to estimate effects. We have shown that the single-species probiotic, a soymilk formulation of *Lactobacillus plantarum* A7, produced multi-biomarker effects on T2D-DN patients without serious adverse events. However, the presence of considerable heterogeneity in evidence still cautions against adopting the clinical use of these nutraceuticals as adjunct therapy. The most promising nutraceutical formulations should be further investigated, in addition to other next-generation options such as postbiotics or FMT, in future clinical trials. In vitro and in vivo studies continue to help us understand the novel and potential mechanisms of action of these nutraceuticals, in turn improving the selection, dosage, and delivery criteria for future clinical trial investigations. In summary, we show that although microbiome-modulating nutraceuticals have shown the potential to alleviate renal health deterioration, overall clinical data does not yet support their unanimous adoption at the bedside, although future trials will help us understand more about their therapeutic potential.

## Figures and Tables

**Table 1:** Studies investigating changes in renal biomarkers following intervention with probiotics, prebiotics, and synbiotics, sorted according to nutraceutical type.

Type of Nutraceutical	Study Design, Country	Participant Demographics * Sample Size and Sex (n, F/M) Age (Mean ± SD; Years) BMI (Mean ± SD; kg/m^2^)		Control/Placebo Substance	Intervention Nutraceutical	Dose × Frequency	Trial Duration	Marker and Effect (If Significant)	Control/Placebo Change ^Φ^	Intervention Change ^Φ^	Overall Effect and Statistical Significance ^Φ^	Author, Year
		Control/Placebo	Intervention									
Probiotic (SS)	PG, DB, RCT (Iran)	T2D-DN n = 20 (10M/10F) 53.6 ± 7.19 26.58 ± 3.27	T2D-DN n = 20 (9M/11F) 56.9 ± 8.1 26.68 ± 3.19	Conventional soy milk	Probiotic soy milk containing *Lactobacillus plantarum* A7 (2 × 10^7^ CFU/mL)	200 mL/d	8 weeks	↓ Serum Cr (mg/dL) **(§)**	C_b_: 1.03 ± 0.16 C_e_: 1.00 ± 0.14 C_Δ_: −0.03 ± 0.08	I_b_: 1.01 ± 0.11 I_e_: 0.83 ± 0.16 I_Δ_: −0.17 ± 0.11 **(I_b_ vs. I_e_ *p* < 0.05) (§)**	**C_Δ_ vs. I_Δ_ (adjusted) *p* < 0.0001 (§)**	[38] χ
								↑ eGFR (mL·min^−1^ (1.73 m^2^)^−1^) **(§)**	C_b_: 72.1 ± 9.1 C_e_: 75.4 ± 11.13 C_Δ_: 3.2 ± 8.4	I_b_: 71.5 ± 9.5 I_e_: 87.5 ± 14.2 I_Δ_: 15.9 ± 10.8 **(I_b_ vs. I_e_ *p* < 0.05) (§)**	**C_Δ_ vs. I_Δ_ (adjusted) *p* < 0.0001 (§)**	
								Serum Phosphorous (mg/dL)	C_b_: 4.38 ± 0.67 C_e_: 4.44 ± 0.59 C_Δ_: 0.05 ± 0.5	I_b_: 4.48 ± 0.47 I_e_: 4.33 ± 0.44 I_Δ_: −0.14 ± 0.10	C_Δ_ vs. I_Δ_ (adjusted) *p* = 0.106 (No significant effect)	
Probiotic (SS)	PG, DB, RCT (Iran)	T2D-DN n = 20 (10M/10F) 53.6 ± 7.19 26.58 ± 3.27	T2D-DN n = 20 (9M/11F) 56.9 ± 8.1 26.68 ± 3.19	Conventional soy milk	Probiotic soy milk containing *Lactobacillus plantarum* A7 (2 × 10^7^ CFU/mL)	200 mL/d	8 weeks	↓ Serum IL-18 (pg/mL) **(§)**	C_b_: 335.14 ± 266.65 C_e_: 326.1 ± 260.34 C_Δ_: −9.03 ± 18.65	I_b_: 286.14 ± 207.8 I_e_: 236.96 ± 181.87 I_Δ_: −49.18 ± 48.22	**C_Δ_ vs. I_Δ_ (adjusted) *p* = 0.002 (§)**	[39] χ
								↓ Urine Alb/Cr (mg/g)	C_b_: 147.0 ± 38.6 C_e_: 141.36 ± 37.9 C_Δ_: −5.7 ± 15.04	I_b_: 145.8 ± 29.1 I_e_: 129.36 ± 31.9 I_Δ_: −16.5 ± 12.2	**C_Δ_ vs. I_Δ_ (adjusted) *p* = 0.03 (§)**	
								↓ Serum sialic acid (mg/dL) **(§)**	C_b_: 232.33 ± 40.79 C_e_: 227.95 ± 40.5 C_Δ_: 4.37 ± 9.91	I_b_: 223.6 ± 44.72 I_e_: 206.2 ± 43.24 I_Δ_: −17.4 ± 11.43	**C_Δ_ vs. I_Δ_ (adjusted) *p* = 0.001 (§)**	
Probiotic (SS)	PG, DB, RCT (Iran)	T2D-DN n = 20 (10M/10F) 53.60 ± 1.60 26.58 ± 0.73	T2D-DN n = 20 (9M/11F) 56.90 ± 1.81 26.68 ± 0.71	Conventional soy milk	Probiotic soy milk containing *Lactobacillus plantarum* A7 (2 × 10^7^ CFU/mL)	200 mL/d	8 weeks	↓ NGAL (ng/mL) (§)	C_b_: 1667.41 ± 420.66 C_e_: 2417.61 ± 392.47 (C_b_ vs. C_e_ *p* = 0.75)	I_b_: 1808.73 ± 510.20 I_e_: 1164.68 ± 379.64 (I_b_ vs. I_e_ *p* = 0.07)	**C_Δ_ vs. I_Δ_ (adjusted) *p* = 0.05 (§)**	[46] χ
								↑ sTNFR1 (ng/mL) (§)	C_b_: 424.80 ± 47.04 C_e_: 348.79 ± 80.89 **(C_b_ vs. C_e_ *p* = 0.04)**	I_b_: 292.53 ± 40.87 I_e_: 353.33 ± 88.02 (I_b_ vs. I_e_ *p* = 0.95)	**C_Δ_ vs. I_Δ_ (adjusted) *p* = 0.03 (§)**	
								↓ Cys-C (ng/mL) (§)	C_b_: 50.40 ± 3.84 C_e_: 58.86 ± 5.44 (C_b_ vs. C_e_ *p* = 0.09)	I_b_: 47.85 ± 2.76 I_e_: 26.82 ± 6.70 (I_b_ vs. I_e_ *p* = 0.12)	**C_Δ_ vs. I_Δ_ (adjusted) *p* = 0.01 (§)**	
								↓ PGRN (ng/mL) (§)	C_b_: 328.85 ± 76.18 C_e_: 399.56 ± 105.20 (C_b_ vs. C_e_ *p* = 0.60)	I_b_: 339.66 ± 109.61 I_e_: 180.90 ± 69.25 (I_b_ vs. I_e_ *p* = 0.83)	**C_Δ_ vs. I_Δ_ (adjusted) *p* = 0.01 (§)**	
Probiotic (SS)	DB, R, PG, PC (Sweden)	T2D-Abdominal obesity n = 15 (11M/4F) 65 ± 5 30.7 ± 4.0	T2D-Abdominal obesity n = 15 (12M/3F) 66 ± 6 30.6 ± 4.5 (low-dose group)	Capsule with mildly sweet tasting powder in an aluminum laminate stick pack	Capsule containing low-dose *Lactobacillus reuteri* DSM 17938 (10^8^ CFU/capsule)	1 capsule/d	12 weeks	Urine Alb/Cr	C_b_: 2.0 ± 2.9 C_e_: 2.2 ± 2.3	I_b_: 2.2 ± 5.9 I_e_: 3.1 ± 8.3	No significant effect	[47] φ
			T2D-Abdominal obesity n = 14 (11M/3F) 64 ± 6 32.3 ± 3.4 (high-dose group)	Capsule with mildly sweet tasting powder in an aluminum laminate stick pack	Capsule containing high-dose *Lactobacillus reuteri* DSM 17938 (10^10^ CFU/capsule)	1 capsule/d	12 weeks	Urine Alb/Cr	C_b_: 2.0 ± 2.9 C_e_: 2.2 ± 2.3	I_b_: 6.7 ± 15.9 I_e_: 6.5 ± 13.4	No significant effect	
Probiotic (SS)	R, DB, CT (Iran)	DN n = 30 (Sex NS) 60.3 ± 8.5 31.1 ± 4.6	DN n = 30 (Sex NS) 62.7 ± 9.1 30.3 ± 5.6	Control honey	Probiotic honey containing viable and heat-resistant *Bacillus coagulans* T4 (10^8^ CFU/g)	25 g/d	12 weeks	BUN (mg/dL)	C_b_: 19.6 ± 6.2 C_e_: 19.9 ± 7.3 C_Δ_: 0.3 ± 4.3	I_b_: 19.6 ± 7.1 I_e_: 19.3 ± 6.8 I_Δ_: 0.3 ± 2.1	C_Δ_ vs. I_Δ_ *p* = 0.54 (No significant effect)	[48]
								Serum Cr (mg/dL)	C_b_: 1.3 ± 0.5 C_e_: 1.5 ± 0.8 C_Δ_: 0.2 ± 0.7	I_b_: 1.6 ± 0.6 I_e_: 1.5 ± 0.5 I_Δ_: −0.1 ± 0.5	C_Δ_ vs. I_Δ_ *p* = 0.09 (No significant effect)	
Probiotic (MS)	R, DB, PC, CT (Iran)	n = 27 (Sex NS) 52.59 ± 7.14 30.17 ± 4.23	n = 27 (Sex NS) 50.51 ± 9.82 31.61 ± 6.36	100 mg fructo-oligosaccharide with lactose/capsule	Freeze-dried *Lactobacillus acidophilus* (2 × 10^9^ CFU), *L. casei* (7 × 10^9^ CFU), *L. rhamnosus* (1.5 × 10^9^ CFU), *L. bulgaricus* (2 × 10^8^ CFU), *Bifidobacterium breve* (2 × 10^10^ CFU), *B. longum* (7 × 10^9^ CFU), *Streptococcus thermophilus* (1.5 × 10^9^ CFU), and 100 mg FOS with lactose carrier per capsule	1 capsule/d	8 weeks	Uric Acid (mg/dL)	C_b_: 4.73 ± 0.27 C_e_: 4.88 ± 0.24 C_Δ_: 0.15 ± 0.21 (C_b_ vs. C_e_ *p* = 0.47)	I_b_: 4.71 ± 0.27 I_e_: 5.51 ± 0.28 I_Δ_: 0.8 ± 0.27 **(I_b_ vs. I_e_ *p* = 0.008) (§)**	C_Δ_ vs. I_Δ_ *p* = 0.07 (No significant effect)	[49]
Probiotic (MS)	DB, R, PG, PC (Malaysia)	n = 68 (34M/34F) 54.2 ± 8.3 29.3 ± 5.3	n = 68 (31M/37F) 52.9 ± 9.2 29.2 ± 5.6	Organoleptically similar sachets without probiotic	Sachets containing viable microbial cell preparation of *Lactobacillus acidophilus*, *L. casei*, *L. lactis*, *Bifidobacterium bifidum*, *B. longum* and *B. infantis* (6.0 × 10^10^ CFU/d total) mixed in water	2 sachets/d	12 weeks	↓ Urea (mmol/L) **(§)**	C_b_: 4.03 ± 0.89 C_1/2_: 4.07 ± 1.10 C_e_: 4.24 ± 1.14 (C_b_ vs. C_e_ *p* = 0.081)	I_b_: 4.26 ± 1.29 I_1/2_: 4.03 ± 1.00 I_e_: 4.04 ± 1.04 (I_b_ vs. I_e_ *p* = 0.086)	**C_Δ_ vs. I_Δ_ (ITT) *p* < 0.05 (§)**	[50]
								Serum Cr (µmol/L)	C_b_: 72.10 ± 18.84 C_1/2_: 71.95 ± 18.60 C_e_: 75.17 ± 18.93 **(C_b_ vs. C_e_ *p* < 0.05) (§)**	I_b_: 69.20 ± 17.36 I_1/2_: 70.87 ± 18.70 I_e_: 72.26 ± 19.73 **(I_b_ vs. I_e_ *p* < 0.05) (§)**	C_Δ_ vs. I_Δ_ (ITT) *p* = 0.329 (No significant effect)	
								eGFR (mL/min)	C_b_: 73.66 ± 13.38 C_1/2_: 73.91 ± 13.58 C_e_: 68.89 ± 13.55 **(C_b_ vs. C_e_ *p* < 0.05) (§)**	I_b_: 74.45 ± 18.5 I_1/2_: 74.14 ± 16.94 I_e_: 73.07 ± 17.13 (I_b_ vs. I_e_ *p* = 0.710)	C_Δ_ vs. I_Δ_ (ITT) *p* = 0.147 (No significant effect)	
								Serum Sodium (mmol/L)	C_b_: 137.9 ± 2.5 C_1/2_: 138.8 ± 2.9 C_e_: 138.5 ± 3.1 (C_b_ vs. C_e_ *p* = 0.167)	I_b_: 138.5 ± 2.2 I_1/2_: 138.9 ± 2.7 I_e_: 138.1 ± 3.5 (I_b_ vs. I_e_ *p* = 0.147)	C_Δ_ vs. I_Δ_ (ITT) *p* = 0.235 (No significant effect)	
								Serum Potassium (mmol/L)	C_b_: 4.40 ± 0.40 C_1/2_: 4.34 ± 0.36 C_e_: 4.37 ± 0.43 (C_b_ vs. C_e_ *p* = 0.360)	I_b_: 4.42 ± 0.30 I_1/2_: 4.42 ± 0.31 I_e_: 4.35 ± 0.31 (I_b_ vs. I_e_ *p* = 0.060)	C_Δ_ vs. I_Δ_ (ITT) *p* = 0.164 (No significant effect)	
Probiotic (MS)	RCT (China)	T2D-DN n = 34 (12M/22F) 56.12 ± 8.23 26.44 ± 2.78	T2D-DN n = 42 (15M/27F) 55.96 ± 8.45 27.51 ± 3.22	Starch	Probiotic supplements containing *Bifidobacterium bifidum*, *Lactobacillus acidophilus*, *Streptococcus thermophilus* (3.2 × 10^9^ CFU/d in total)	1 capsule/d	12 weeks	↓ Urine Alb/Cr (mg/g) **(§)**	C_b_: 99. 66 ± 25.24 C_e_: 87.71 ± 23.01	I_b_: 101.60 ± 22.17 I_e_: 67.53 ± 20.11	**C_e_ vs. I_e_ *p* < 0.05 (§)**	[51]
								eGFR (ml·min^−1^ (1.73 m^2^) ^−1^)	C_b_: 83.12 ± 7.2 C_e_: 84.28 ± 7.13 (C_b_ vs. C_e_ *p* = 0.77)	I_b_: 82.8 ± 8.72 I_e_: 84.34 ± 6.97 (I_b_ vs. I_e_ *p* = 0.45)	C_e_ vs. I_e_ *p* = 0.08 (No significant effect)	
Probiotic (MS)	R, DB, PC (Iran)	DN n = 30 (28/30 = T2D) (2/30 = T1D) Sex NS 60.9 ± 4.4 26.3 ± 3.2	DN n = 30 (28/30 = T2D) (2/30 = T1D) Sex NS 58.9 ± 8.8 25.3 ± 2.3	Starch	Probiotic supplements with *Lactobacillus acidophilus* ZT-L1, *Bifidobacterium bifidum* ZT-B1, *L. reuteri* ZT-Lre, and *L. fermentum* ZT-L3 (8 × 10^9^ CFU/d in total)	1 capsule/d	12 weeks	↓ BUN (mg/dL) **(§)**	C_b_: 22.2 ± 9.9 C_e_: 22.6 ± 12.1 C_Δ_: 0.4 ± 7.7	I_b_: 23.5 ± 10.6 I_e_: 20.0 ± 8.3 I_Δ_: −3.5 ± 5.8	**C_Δ_ vs. I_Δ_ *p* = 0.03 (§)**	[52]
								↓ Serum Cr (mg/dL) **(§)**	C_b_: 1.3 ± 0.5 C_e_: 1.4 ± 0.5 C_Δ_: 0.1 ± 0.2	I_b_: 1.5 ± 0.5 I_e_: 1.3 ± 0.5 I_Δ_: −0.2 ± 0.3	**C_Δ_ vs. I_Δ_ *p* = 0.001 (§)**	
								↑ eGFR (mL/min) **(§)**	C_b_: 68.4 ± 25.1 C_e_: 65.1 ± 24.1 C_Δ_: −3.2 ± 6.4	I_b_: 58.4 ± 22.8 I_e_: 66.7 ± 25.8 I_Δ_: 8.3 ± 17.3	**C_Δ_ vs. I_Δ_ *p* = 0.001 (§)**	
								Urine Protein (mg/day)	C_b_: 1330.0 ± 637 C_e_: 1331.3 ± 640 C_Δ_: 0.13 ± 33.5	I_b_: 1261.7 ± 698.3 I_e_: 1247.3 ± 713.4 I_Δ_: −14.3 ± 40.1	C_Δ_ vs. I_Δ_ *p* = 0.10 (No significant effect)	
Probiotic (MS)	R, DB, PC, CT (Iran)	Diabetic hemodialysis n = 30 (20M/10F) (27/30 = T2D) (3/30 = T1D) 59.4 ± 16.0 27.0 ± 6.4	Diabetic hemodialysis n = 30 (20M/10F) (27/30 = T2D) (3/30 = T1D) 54.0 ± 16.0 25.5 ± 5.6	‘Placebo’	Probiotic capsule containing *Lactobacillus acidophilus, L. casei*, and *Bifidobacterium bifidum* (2 × 10^9^ CFU/g each)	1 capsule/d	12 weeks	eGFR (ml·min^−1^ (1.73 m^2^)^−1^)	C_b_: 2.22 ± 0.86 C_e_: 2.25 ± 0.93 C_Δ_: 0.02 ± 0.20 (C_b_ vs. C_e_ *p* = 0.46)	I_b_: 2.49 ± 1.15 I_e_: 2.54 ± 1.16 I_Δ_: 0.04 ± 0.18 (I_b_ vs. I_e_ *p* = 0.23)	C_Δ_ vs. I_Δ_ *p* = 0.77; adjusted *p* = 0.74 (No significant effect)	[53]
								Serum Cr (mg/dL)	C_b_: 7.8 ± 3.0 C_e_: 7.7 ± 2.9 C_Δ_: −0.1 ± 0.8 (C_b_ vs. C_e_ *p* = 0.48)	I_b_: 7.4 ± 3.1 I_e_: 7.2 ± 2.6 I_Δ_: −0.2 ± 1.2 (I_b_ vs. I_e_ *p* = 0.39)	C_Δ_ vs. I_Δ_ *p* = 0.73; adjusted *p* = 0.33 (No significant effect)	
								BUN (mg/dL)	C_b_: 53.6 ± 19.5 C_e_: 52.3 ± 12.7 C_Δ_: −1.3 ± 16.1 (C_b_ vs. C_e_ *p* = 0.65)	I_b_: 64.9 ± 29.5 I_e_: 63.9 ± 26.0 I_Δ_: −1.0 ± 32.6 (I_b_ vs. I_e_ *p* = 0.85)	C_Δ_ vs. I_Δ_ *p* = 0.96; adjusted *p* = 0.17 (No significant effect)	
								Serum Albumin (g/dL)	C_b_: 4.0 ± 0.4 C_e_: 4.1 ± 0.4 C_Δ_: 0.1 ± 0.4 (C_b_ vs. C_e_ *p* = 0.38)	I_b_: 4.2 ± 0.4 I_e_: 4.3 ± 0.4 I_Δ_: 0.1 ± 0.3 (I_b_ vs. I_e_ *p* = 0.45)	C_Δ_ vs. I_Δ_ *p* = 0.84; adjusted *p* = 0.48 (No significant effect)	
								Serum Sodium (mmol/L)	C_b_: 137.1 ± 4.2 C_e_: 138.1 ± 2.9 C_Δ_: 1.0 ± 3.8 (C_b_ vs. C_e_ *p* = 0.10)	I_b_: 135.9 ± 3.3 I_e_: 136.2 ± 3.1 I_Δ_: 0.3 ± 3.8 (I_b_ vs. I_e_ *p* = 0.63)	C_Δ_ vs. I_Δ_ *p* = 0.51; adjusted *p* = 0.07 (No significant effect)	
								Serum Potassium (mmol/L)	C_b_: 4.6 ± 0.7 C_e_: 4.4 ± 0.4 C_Δ_: −0.1 ± 0.6 (C_b_ vs. C_e_ *p* = 0.10)	I_b_: 4.8 ± 0.6 I_e_: 4.7 ± 0.7 I_Δ_: −0.1 ± 0.6 (I_b_ vs. I_e_ *p* = 0.19)	C_Δ_ vs. I_Δ_ *p* = 0.87; adjusted *p* = 0.18 (No significant effect)	
Probiotic (MS)	SB, PC, CT (Egypt)	Diabetic ESRD hemodialysis n = 30 (18M/12F) 50.9 ± 16.9 BMI NS	Diabetic ESRD hemodialysis n = 30 (12M/18F) 57.7 ± 11.4 BMI NS	Placebo capsules, ESA, and anti-diabetic agents	Capsules containing study agent (5 × 10^6^ of *Lactobacillus delbrueckii* and *L. fermentum*), ESA, and antidiabetic agents	1 capsule/d	12 weeks	Serum Albumin (g/dL)	C_b_: 3.5 (IQR: 3.1−4.0) C_e_: 3.5 (IQR: 3.0–3.6) (C_b_ vs. C_e_ *p* = 0.116)	I_b_: 3.4 (IQR: 3.2–3.5) I_e_: 3.5 (IQR: 3.1–3.8) **(I_b_ vs. I_e_ *p* = 0.039) (§)**	Effect NS	[40]
Prebiotic	DB, PC (Iran)	T2D-Overwight n = 22 (22F) 48.61 ± 9.16 29.98 ± 4.01	T2D-Overwight n = 27 (27F) 48.07 ± 8.70 31.43 ± 3.50	Maltodextrin	Oligofructose-enriched chicory inulin	5 × 2 g/d	2 months	Serum Cr (mg/dL)	C_b_: 0.78 ± 0.09 C_e_: 0.82 ± 0.14 (C_b_ vs. C_e_ *p* = 0.16)	I_b_: 0.77 ± 0.11 I_e_: 0.79 ± 0.10 (I_b_ vs. I_e_ *p* = 0.47)	C_e_ vs. I_e_ *p* = 0.44 (No significant effect)	[41]
								Serum Phosphorous (mg/dL)	C_b_: 4.23 ± 0.45 C_e_: 4.00 ± 0.45 **(C_b_ vs. C_e_ *p* = 0.013)**	I_b_: 3.96 ± 0.48 I_e_: 3.96 ± 0.56 (I_b_ vs. I_e_ *p* = 0.97)	C_e_ vs. I_e_ *p* = 0.80 (No significant effect)	
								eGFR (ml·min^−1^ (1.73 m^2^)^−1^)	C_b_: 85.30 ± 13.45 C_e_: 82.05 ± 16.06 (C_b_ vs. C_e_ *p* = 0.28)	I_b_: 86.34 ± 13.96 I_e_: 84.30 ± 13.57 (I_b_ vs. I_e_ *p* = 0.44)	C_e_ vs. I_e_ *p* = 0.65 (No significant effect)	
Prebiotic	R, PC, TB, CT (Iran)	T2D-Overwight n = 33 (33F) 48.6 ± 7.9 32.0 ± 3.9	T2D-Overwight n = 32 (32F) 49.5 ± 8.0 31.5 ± 4.5	Maltodextrin	Resistant dextrin supplement (NUTRIOSE^®^06)	5 × 2 g/d	8 weeks	Uric Acid (mg/dL)	C_b_: 5.40 ± 0.61 C_e_: 5.50 ± 0.33 C_Δ_ MD: 0.10 (95% CI: −0.80; 1.12) (C_b_ vs. C_e_ *p* = 0.28)	I_b_: 4.80 ± 0.40 I_e_: 5.60 ± 0.20 I_Δ_ MD: 1.85 (95% CI: 0.91; 1.24) **(I_b_ vs. I_e_ *p* < 0.05) (§)**	C_Δ_ vs. I_Δ_ MD: 0.10 (95% CI: −1.55; 0.75) (No significant effect)	[42]
Prebiotic	R, DB, PC (Japan)	n = 25 (17M/8F) 54 ± 12 27.2 ± 4.6	n = 27 (21M/6F) 55 ± 11 27.9 ± 3.6	Maltodextrin syrup	GOS syrup (Oligomate55N)	10 g/d	4 weeks	BUN (mg/dL)	C_b_: 14.0 ± 4.0 C_e_: 13.0 ± 3.0 (C_b_ vs. C_e_ *p* > 0.05)	I_b_: 15.0 ± 5.0 I_e_: 15.0 ± 5.0 (I_b_ vs. I_e_ *p* > 0.05)	No significant effect	[43]
								Serum Cr (mg/dL)	C_b_: 0.7 ± 0.2 C_e_: 0.8 ± 0.2 **(C_b_ vs. C_e_ *p* < 0.05) (§)**	I_b_: 0.9 ± 0.5 I_e_: 0.9 ± 0.4 (I_b_ vs. I_e_ *p* > 0.05)	No significant effect	
								eGFR (mL/min)	C_b_: 85.1 ± 21.3 C_e_: 79.9 ± 18.7 **(C_b_ vs. C_e_ *p* < 0.05) (§)**	I_b_: 75.1 ± 24.4 I_e_: 71.2 ±2 1.6 **(I_b_ vs. I_e_ *p* < 0.05) (§)**	Effect NS	
Synbiotic (SS)	R, DB, CC, CT (Iran)	n = 62 (19M/43F) 53.1 ± 8.7 29.90 ± 5.18	n = 62 (19M/43F) 53.1 ± 8.7 29.60 ± 4.53	0.38 g isomalt, 0.36 g sorbitol and 0.05 g stevia per 1g	Heat-resistant *Lactobacillus sporogenes* (1 × 10^7^ CFU), 0.04 g inulin, 0.38 g isomalt, 0.36 g sorbitol and 0.05 g stevia per gram	9 × 3 g/d	6 × 2 weeks	↑ Uric acid (mg/dL) (§)	C_b_: 5.5 ± 0.3 C_e_: 5.4 ± 0.2 C_Δ_: −0.1 ± 0.3	I_b_: 4.9 ± 0.2 I_e_: 5.6 ± 0.2 I_Δ_: 0.7 ± 0.2	**C_Δ_ vs. I_Δ_ *p* = 0.03 (§)**	[44] ψ
Synbiotic (MS)	SC, R, DB, PC (Iran)	n = 35 (19M/16F) 58.63 ± 8.06 27.30 ± 3.81	n = 35 (23M/12F) 58.71 ± 8.20 28.13 ± 3.78	Capsules containing row starch, B group vitamins (1 mg), lactose (0.5 mg), malt-dextrin, magnesium saturate and talc	Capsules containing *Lactobacillus* family, *Bifidobacterium* family, *Streptococcus thermophilus*, FOS, B group vitamins (1 mg), lactose (0.5 mg), maltodextrin, magnesium saturate and talc	1 × 500 mg capsule/d	9 weeks	Urea (mg/dL)	C_b_: 36.80 ± 14.79 C_e_: 37.94 ± 14.57 C_Δ_: −1.14 ± 7.30 (C_b_ vs. C_e_ *p* = 0.36)	I_b_: 31.20 ± 7.67 I_e_: 33.25 ± 7.61 I_Δ_: −2.05 ± 7.31 (I_b_ vs. I_e_ *p* = 0.10)	C_e_ vs. I_e_ *p* = 0.09 C_Δ_ vs. I_Δ_ *p* = 0.60 (No significant effect)	[45]
								Serum Cr (mg/dL)	C_b_: 1.05 ± 0.22 C_e_: 1.03 ± 0.24 C_Δ_: 0.02 ± 0.11 (C_b_ vs. C_e_ *p* = 0.22)	I_b_: 1.04 ± 0.26 I_e_: 1.05 ± 0.26 I_Δ_: −0.00 ± 0.09 (I_b_ vs. I_e_ *p* = 0.82)	C_e_ vs. I_e_ *p* = 0.73 C_Δ_ vs. I_Δ_ *p* = 0.73 (No significant effect)	
								↓ Urine Alb/Cr (mg/g) **(§)**	C_b_: 62.77 ± 59.6 C_e_: 81.09 ± 81.58 C_Δ_: 18.31 ± 46.78 **(C_b_ vs. C_e_ *p* = 0.027) (§)**	I_b_: 45.39 ± 38.85 I_e_: 34.94 ± 13.1 I_Δ_: −10.44 ± 35.26 (I_b_ vs. I_e_ *p* = 0.089)	**C_e_ vs. I_e_ *p* = 0.00 (§)** **C_Δ_ vs. I_Δ_ *p* < 0.0001** **(§)**	

* All participants are diagnosed with diabetes; other inclusionary comorbidities, if specified, have also been mentioned; **^Φ^** Values provided as mean ± standard deviation (**SD**), mean difference (**MD**) and 95% confidence interval (**CI**), or median and interquartile range (**IQR**); **(§)** denotes statistically significant results; **χ** [38,39,46] are derived from the same clinical trial and involve the same participants; hence, they have been considered one RCT. **φ** [47] consists of two intervention groups linked to the same control/placebo group and considered as two RCTs; however, the number of control participants has not been considered twice. **Ψ** [44] is a crossover-controlled trial; hence, the number of total participants has been considered as 62; Abbreviations: **BMI =** body mass index; **F** = female; **M** = male; **ITT =** intention-to-treat analysis; **C_b_** = control group baseline value; **C_e_** = control group end-of-trial value; **C_Δ_**= control group value change over trial duration; **C_1/2_**= control group value change over half trial duration; **I_b_** = intervention group baseline value; **I_e_** = intervention group end-of-trial value; **I_Δ_**= control group value change over trial duration; **I_1/2_**= intervention group value change over half-trial; **§** = significant effect; **T1D** = type 1 diabetes; **T2D** = type 2 diabetes; **ESRD =** end-stage renal disease; **DN =** diabetic nephropathy; **NS** = not specified; **SS =** single species; **MS** = multispecies; **SB** = single-blinded; **DB** = double-blinded; **TB** = triple-blinded; **R** = randomized; **RCT** = randomized controlled trial; **CC** = crossover-controlled; **PC** = placebo-controlled; **PG** = parallel group; **CT** = clinical trial; **ESA** = erythropoietin stimulating agent; **BUN =** blood urea nitrogen; **eGFR** = estimated glomerular filtration rate; **IL-18** = interleukin-18; **PGRN** = progranulin; **sTNFR1** = a cytokine receptor soluble tumor necrosis factor receptor; **NGAL** = neutrophil gelatinase-associated lipocalin; **Cys-C** = cystatin C; **Cr** = creatinine; **Alb/Cr**: albumin/creatinine ratio; **FOS** = fructo-oligosaccharide, **GOS** = galacto-oligosaccharide; **CFU** = colony forming units.

## Data Availability

Not applicable as no new data were created in this project; the extracted data templates can be requested from the corresponding authors.

## Supplementary Materials

The following supporting information can be downloaded at: https://www.mdpi.com/article/10.3390/ijms232314838/s1.
