# Supplementary material for: Renal Health Improvement in Diabetes through Microbiome Modulation of the Gut–Kidney Axis with Biotics: A Systematic and Narrative Review of Randomized Controlled Trials
Source: Int J Mol Sci. 2022 Nov 27;23(23):14838. doi: 10.3390/ijms232314838 (PMC9740604; doi:10.3390/ijms232314838)
Supplement: Supplementary file 1 [file ijms-23-14838-s001.zip › ijms-2028621-supplementary.pdf]

Supplementary Table S1. Detailed Search Strategy in Each Database.

Database	Search Strategy (Last Updated: 10 April 2022)
**PubMed**	("Probiotics"[MeSH Terms] OR "probiotics"[Title/Abstract] OR "probiotic"[Title/Abstract] OR "Prebiotics"[MeSH Terms] OR "prebiotic"[Title/Abstract] OR "prebiotics"[Title/Abstract] OR "Synbiotics"[MeSH Terms] OR "synbiotics"[Title/Abstract] OR "synbiotic"[Title/Abstract] OR "symbiotic"[Title/Abstract] OR "symbiotics"[Title/Abstract] OR "gastrointestinal microbiome"[MeSH Terms] OR "gut microbiome"[Title/Abstract] OR "gut flora"[Title/Abstract]) AND ("diabetes mellitus, type 2"[MeSH Terms] OR "T2D"[Title/Abstract] OR "type 2 diabetes"[Title/Abstract]). Limit to clinical and animal studies.
**Scopus**	(INDEXTERMS ("clinical trials" OR "clinical trials as a topic" OR "randomized controlled trial" OR "Randomized Controlled Trials as Topic" OR "controlled clinical trial" OR "Controlled Clinical Trials" OR "random allocation" OR "Double-Blind Method" OR "Single-Blind Method" OR "Cross-Over Studies" OR "Placebos" OR "multicenter study" OR "double blind procedure" OR "single blind procedure" OR "crossover procedure" OR "clinical trial" OR "controlled study" OR "randomization" OR "placebo")) OR (TITLE-ABS-KEY (("clinical trials" OR "clinical trials as a topic" OR "randomized controlled trial" OR "Randomized Controlled Trials as Topic" OR "controlled clinical trial" OR "Controlled Clinical Trials as Topic" OR "random allocation" OR "randomly allocated" OR "allocated randomly" OR "Double-Blind Method" OR "Single-Blind Method" OR "Cross-Over Studies" OR "Placebos" OR "cross-over trial" OR "single blind" OR "double blind" OR "factorial design" OR "factorial trial"))) OR (TITLE-ABS (clinical AND trial* OR rct* OR random* OR blind*)) AND (((TITLE-ABS-KEY (probiotics OR probiotic OR prebiotic OR prebiotics)) OR (TITLE-ABS-KEY (synbiotics OR synbiotic OR symbiotic OR symbiotics)) OR TITLE-ABS-KEY ("gastrointestinal microbiome") OR TITLE-ABS-KEY ("gut microbiome") OR TITLE-ABS-KEY ("gut flora") AND (TITLE-ABS-KEY (diabetes AND mellitus AND type 2) OR TITLE-ABS-KEY (t2d) OR TITLE-ABS-KEY (type 2 diabetes))))
**Web of Science**	TOPIC ((probiotic* OR prebiotic* OR sy*biotic OR symbiotic* OR synbiotic*) OR ("gastrointestinal microbiome" OR "gut microbiome" OR "gut flora")) AND TOPIC ("diabetes mellitus type 2" OR t2d OR "type 2 diabetes"). Limit to: Clinical Trials.
**Embase**	(probiotic*.mp. [mp=title, abstract, heading word, drug trade name, original title, device manufacturer, drug manufacturer, device trade name, keyword, floating subheading word, candidate term word] OR prebiotic*.mp. [mp=title, abstract, heading word, drug trade name, original title, device manufacturer, drug manufacturer, device trade name, keyword, floating subheading word, candidate term word] OR sy*biotics.mp. [mp=title, abstract, heading word, drug trade name, original title, device manufacturer, drug manufacturer, device trade name, keyword, floating subheading word, candidate term word] OR sy*biotic.mp. [mp=title, abstract, heading word, drug trade name, original title, device manufacturer, drug manufacturer, device trade name, keyword, floating subheading word, candidate term word] OR gut flora.mp. [mp=title, abstract, heading word, drug trade name, original title, device manufacturer, drug manufacturer, device trade name, keyword, floating subheading word, candidate term word] OR gastrointestinal microbiome.mp. [mp=title, abstract, heading word, drug trade name, original title, device manufacturer, drug manufacturer, device trade name, keyword, floating subheading word, candidate term word] OR gut microbiome.mp. [mp=title, abstract, heading word, drug trade name, original title, device manufacturer, drug manufacturer, device trade name, keyword, floating subheading word, candidate term word]) AND (diabetes type 2.mp. [mp=title, abstract, heading word, drug trade name, original title, device manufacturer, drug manufacturer, device trade name, keyword, floating subheading word, candidate term word] OR t2d.mp. [mp=title, abstract, heading word, drug trade name, original title, device manufacturer, drug manufacturer, device trade name, keyword, floating subheading word, candidate term word] OR type 2 diabetes.mp. [mp=title, abstract, heading word, drug trade name, original title, device manufacturer, drug manufacturer, device trade name, keyword, floating subheading word, candidate term word] OR diabetes mellitus.mp. [mp=title, abstract, heading word, drug trade name, original title, device manufacturer, drug manufacturer, device trade name, keyword, floating subheading word, candidate term word]. Limit to (clinical trial or randomized controlled trial or controlled clinical trial or multicenter study or phase 1 clinical trial or phase 2 clinical trial or phase 3 clinical trial or phase 4 clinical trial)

**Clinical Trials**	("Diabetes Mellitus, Type 2" OR "Type 2 diabetes" OR Diabetes) AND (prebiotic OR probiotic OR symbiotic OR synbiotic OR "gastrointestinal microbiome" OR "Gut Microbiota"). Filtered by Completed Studies	
**ProQuest Dissertations and Theses**	(Probiotic* OR Prebiotic* OR Synbiotic* OR Symbiotic* OR "gastrointestinal microbiome" OR "Gut Flora" OR "gut microbiome") AND ab(diabetes AND ("type 2" OR t2d OR "Type II"))	
**Cochrane**	1 MeSH descriptor: [Probiotics]	12 "gut flora"
	2 probiotic	13 "gastrointestinal microbiome"
	3 prebiotic	14 "gut microbiome"
	4 MeSH descriptor: [Prebiotics]	15 "diabetes mellitus"
	5 symbiotic	16 MeSH descriptor: [Diabetes Mellitus]
	6 synbiotic	17 t2d
	7 MeSH descriptor: [Synbiotics]	18 "type 2" AND diabetes
	8 probiotics	19 1 or 2 or 3 or 4 or 5 or 6 or 7 or 8 or 9 or 10 or 11 or 12 or 13 or 14
	9 prebiotics	20 15 or 16 or 17 or 18
	10 sy*biotics	21 19 and 20
	11 MeSH descriptor: [Gastrointestinal Microbiome]	22 Limit to: Controlled trials

Supplementary Table S2. Data Extraction Variables

**1.**	**Study characteristics**	First author's last name, year of publication, country of study, primary outcomes, study design, trial duration and investigated biomarker(s) (baseline, end-of-trial, mid-trial, change over time)
**2.**	**Participant characteristics**	Mean and standard deviation (SD) of age and baseline body mass index (BMI), ratio and number of participants sexes, total number of participants, presence of inclusionary comorbidities for both intervention and placebo/control groups.
**3.**	**Intervention characteristics**	Type, composition and daily dosage of nutraceutical and control/placebo substance.

Supplementary Figure S1. Summary of risk of bias assessment for the included studies.

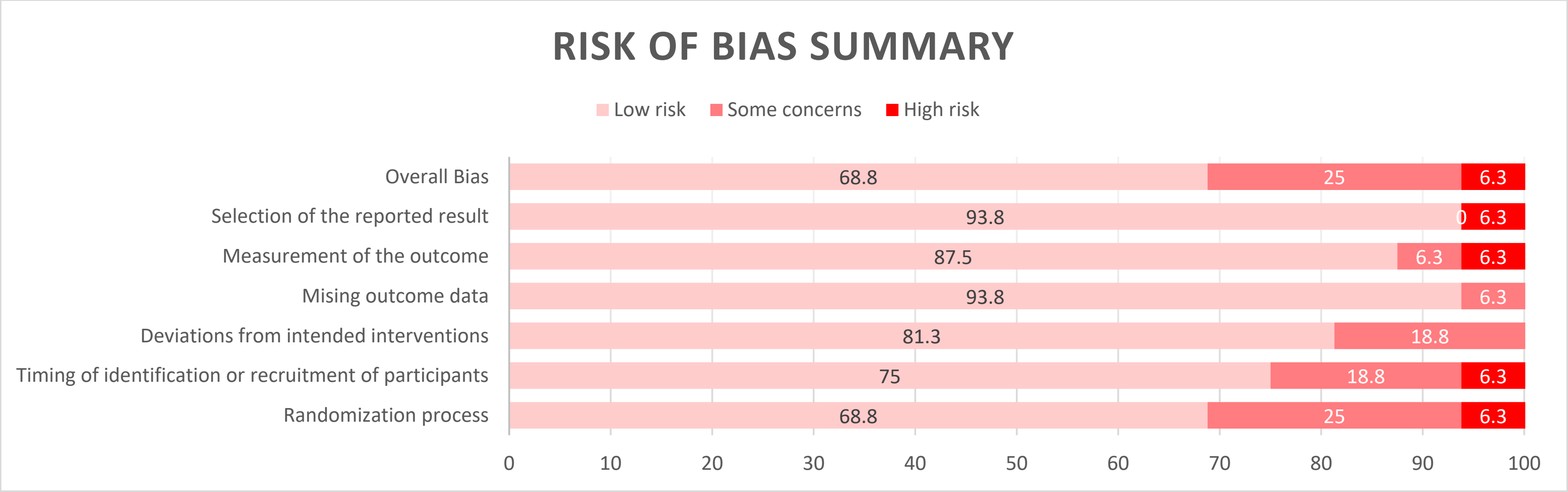

Supplementary Figure S2. Individual study risk of bias assessments.

Author, year	D1a	D1b	D2	D3	D4	D5	Overall
Mobini, 2017	+	+	+	+	+	+	+
Mazruei Arani, 2019	!	!	+	+	+	+	!
Firouzi, 2015	!	+	+	+	+	+	+
Mafi, 2018	!	!	!	!	+	+	!
Soleimani, 2017	+	+	+	+	+	+	+
Mosbah, 2020	-	-	!	+	-	-	-
Farhangi, 2016	+	+	+	+	+	+	+
Farhangi, 2020	+	+	+	+	+	+	+
Gonai, 2017	!	+	+	+	+	+	!
Asemi, 2014	+	+	+	+	+	+	+
Ebrahimi, 2017	+	+	+	+	+	+	+
Asemi, 2013	+	+	+	+	+	+	+
Abbasi, 2017	+	+	+	+	+	+	+
Abbasi, 2018	+	+	+	+	+	+	+
Jiang, 2021	+	!	!	+	!	+	!
Miraghajani, 2019	+	+	+	+	+	+	+

+ Low risk
! Some concerns
- High risk

D1a	Randomisation process
D1b	Timing of identification or recruitment of participants
D2	Deviations from the intended interventions
D3	Missing outcome data
D4	Measurement of the outcome
D5	Selection of the reported result
